# Evaluation of carbonyl collection methods in electronic cigarette aerosols

**DOI:** 10.3389/fchem.2025.1694858

**Published:** 2025-11-26

**Authors:** Bianca Martinez, Yeongkwon Son, Andrey Khlystov

**Affiliations:** Organic Analytical Laboratory, Division of Atmospheric Science, Desert Research Institute, Reno, NV, United States

**Keywords:** electronic cigarettes, e-cigarettes, carbonyl compounds, formaldehyde, acetaldehyde, carbonyl sampling methods evaluation, impingers method, DNPH cartridges

## Abstract

**Introduction:**

This study evaluated the performance of three carbonyl collection media that are frequently used in electronic cigarette (e-cig) studies, namely: 2,4-Dinitrophenyl hydrazine (DNPH) silica impregnated cartridges (C), a DNPH-coated glass fiber filter (DF), and an impinger with a DNPH solution (I).

**Methods:**

These collection methods were tested with a ‘mod’ and a ‘pod’ e-cigs that represent devices producing different amounts of e-cig aerosols. The tests were performed using two different puff topography regimes to investigate the effect of puff flow rate on carbonyl collection efficiency. Carbonyls emitted from the e-cigs were collected using I-I (two impingers in series), C (a single DNPH cartridge), and DF-C (a DNPH filter followed by a DNPH cartridge). Samples were analyzed using high-performance liquid chromatography coupled with photodiode array detector.

**Results:**

Measured carbonyl levels varied between the three methods. For both devices, the highest values were measured with C and DF-C. Results from the ‘pod’ device measured with I-I were comparable with the other methods, but lower than the other two methods for the ‘mod’ device. For example, 1 LPM ‘mod’ acetaldehyde emissions measured with C (0.09 ± 0.02 µg/puff) and DF-C (0.14 ± 0.09 µg/puff) were significantly higher (p = 0.06 and 0.04, respectively) than I-I method (0.05 ± 0.02 µg/puff).

**Discussion:**

The likely cause for the underestimation by the I-I method is the inefficient collection of particle phase bound carbonyls by the impingers and, possibly, wall losses in the inlet of the impinger. Based on our results, C provides the best approach for carbonyl collection in e-cig aerosols, while I-I that is used in the standard testing method can underestimate carbonyl emissions when measuring high concentration aerosols such as those produced by ‘mod’ devices.

## 1 Introduction

Electronic cigarettes (e-cigarettes) were introduced as safer alternatives to combustible tobacco products (Gulsen and Uslu, 2020). Early advertisements for e-cigarettes emphasized their advantages over traditional cigarettes, targeting users of combustible tobacco products. In contrast, newer versions highlight various flavors and product versatility (Unger and Unger, 2018; Zhu et al., 2014). Since their introduction, e-cigarettes have rapidly gained popularity, and diverse styles have been released onto the market (Collins et al., 2019; Madison et al., 2019). E-cigarettes are available in various formats (e.g., cig-a-like, mod, and pod), equipped with different coil materials (*e.g.,* Kanthal [FeAlCr alloy], nichrome [NiCr alloy], titanium), wick materials (*e.g.,* cotton and silica), and provide different power outputs. They also offer a wide range of e-liquid compositions, including different flavorings, nicotine content, and base material composition (propylene glycol to vegetable glycerin ratios) (Son et al., 2020a). Currently, pod devices are the most popular (51.8% in December 2022) (Ali et al., 2023). The main difference between pod and mod devices lies in their aerosol output. Mod devices, which have larger battery boxes and tanks, produce significantly larger amounts of aerosols than pod devices (Hilpert et al., 2021; Son et al., 2020b).

Public health concerns arose as e-cigarettes were shown to emit dangerous amounts of formaldehyde and other carbonyl compounds (Jensen et al., 2015; Khlystov and Samburova, 2016). This is especially concerning because e-cigarettes became the most popular tobacco product among youth in the past decade (7.7% of high and middle school students in 2023), while traditional cigarettes became one of the least popular products (1.6%) (Birdsey et al., 2023). Thus, a precise analysis of harmful constituents emitted by e-cigarettes is necessary to understand the impact of e-cigarette use on public health and to help establish effective regulations.

Accurate measurements of toxic chemical compounds emitted by various e-cigarette products are challenging due to the variabilities listed above. For example, differences in aerosol formation capacity and e-liquid composition can lead to variations in the emission of harmful compounds (*e.g.,* formaldehyde and acetaldehyde) (Geiss et al., 2015; Khlystov and Samburova, 2016; Kosmider et al., 2014; Olmedo et al., 2016; Son et al., 2020b). Different mass spectrometry (MS)-based methods were reported for measurements of volatile organic compounds (VOCs) in mainstream and secondary e-cigarette emissions, as well as their content in e-liquids (Augustini et al., 2023; LeBouf et al., 2018; Patel et al., 2021; Valenzuela et al., 2025). However, measurements of carbonyl compounds are difficult with MS techniques as these compounds are difficult to ionize (Sun et al., 2021). Instead, collection and analysis methods that use 2,4-Dinitrophenylhydrazine (DNPH) are most commonly used to sample these compounds in mainstream e-cigarette aerosols (Flora et al., 2017; Jin et al., 2021; Lee et al., 2018).

The reported levels of toxic carbonyls emitted by e-cigarettes have discrepancies, likely due to different testing methods used by researchers and industries (Farsalinos et al., 2015; Geiss et al., 2015; Kosmider et al., 2014; Talih et al., 2016; Tayyarah and Long, 2014). Numerous e-cigarette studies and testing laboratories have used the impinger method (Chen et al., 2021; Farsalinos et al., 2015; Farsalinos et al., 2018; Farsalinos and Voudris, 2018; Gillman et al., 2016), which was developed for conventional cigarette testing. The impinger method was designed to capture gaseous carbonyls in combustible tobacco smoke (CORESTA, 2021). Studies using this method have reported lower carbonyl emissions than those using alternative testing methods (*e.g.,* filter and cartridge methods) that capture both gaseous and particle phases (Geiss et al., 2015; Geiss et al., 2016; Jensen et al., 2015; Khlystov and Samburova, 2016; Salamanca et al., 2018; Son et al., 2020b; Uchiyama et al., 2016). The particle phase could contain a large fraction of carbonyls in the e-cigarette aerosols (Jensen et al., 2015; Salamanca et al., 2018; Uchiyama et al., 2016). However, systematic comparisons of carbonyl testing methods remain unreported, as do investigations of how puff topography, aerosol loading, or base e-liquid constituents affect method performance.

To address this knowledge gap, this study evaluates three carbonyl collection methods often used to test e-cigarette mainstream carbonyl emissions: a series of impingers containing a DNPH solution (I-I), a DNPH cartridge (C), and a DNPH filter followed by a DNPH cartridge (DF-C). These methods were tested by sampling aerosols produced by mod- and pod-type e-cigarettes.

## 2 Materials and methods

### 2.1 Chemicals

The following chemicals were used: formalin (37% formaldehyde; CAS: 50-00-0, 7732-18-5; purity: 34.5%, Fisher Chemical, Fair Lawn, NJ, United States), acetonitrile (high-performance liquid chromatography [HPLC] grade; CAS: 75-05-8; EMD Millipore Corp, Billerica, MA, United States), tetrahydrofuran (non-UV, HPLC-grade, 99.7%, stab with 250 ppm BHT; CAS: 109-99-9, Alfa Aesar, Ward Hill, MA, United States), propylene glycol (PG; 1,2-propaneidiol, ACS, 99.5%; CAS: 57-55-6, Thermo Fisher Scientific, Ward Hill, MA, United States), glycerol (VG; Glycerol, 99%+; CAS: 56-81-5, Thermo Fisher Scientific, Ward Hill, MA, United States), perchloric acid (OmniTrace ^®^ 65%–71%; CAS: 7,601-90-3, EMD Millipore Comp., Billerica, MA, United States), a certified carbonyl calibration mixture (TO11/IP-6A aldehyde/ketone–DNPH Mix, CRM4M7285; formaldehyde-DNPH, acetaldehyde-DNPH, acetone-DNPH, acrolein-DNPH, propionaldehyde-DNPH, crotonaldehyde-DNPH, n-butyraldehyde-DNPH, benzaldehyde-DNPH, isovaleraldehyde-DNPH, valeraldehyde-DNPH, O–tolualdehyde-DNPH, M + P-tolualdehyde-DNPH, hexaldehyde-DNPH, and 2,5-dimethylbenzaldehyde-DNPH, Sigma Aldrich, Laramie, WY, United States), DNPH-silica cartridges (Sep-Pak ^®^ DNPH-Silica Cartridges Plus-[350 mg], part No.: WAT047205, Waters, Milford, MA, United States), and 37 mm DNPH-coated glass fiber filters (ORBO 827 LpDNPH Coated Filter, Supelco, Folsom, CA, United States). For a 9 mM acidified DNPH solution (pH = 2–3), 2.54 g of DNPH reagent (CAS: 119-26-6; 100 g; Spectrum Chemical; MFC Corp., Gardena, CA) were dissolved in 5 mL of perchloric acid and diluted with acetonitrile for a final volume of 1.00 L.

### 2.2 Electronic cigarette aerosol generation

**FIGURE 1:**
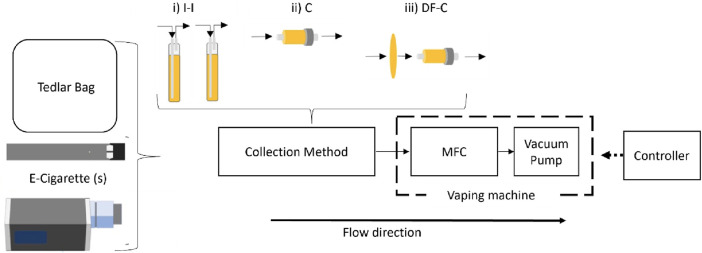
Vaping machine diagram for e-cigarette aerosol collection.

E-cigarette aerosols were generated using a custom-made vaping machine. The vaping machine consisted of a mass flow controller (MFC, MC-2SLPM-D/5M, Alicat Scientific, Tucson, AZ), solenoid valves, and a vacuum pump (see Figure 1). The MFC and valves were controlled using a Python program via a LabJack T7 controller (LabJack Co., Lakewood, CO, United States) to achieve the desired puff duration, flow rate, and number of puffs.

Mod- and pod-type devices were used to generate e-cigarette aerosols. A mod SCAR-18 (0.4-Ω, nichrome coil, 50-W power, manufacturer: SMOK) and a strawberry–watermelon-flavored e-liquid (Brand: Candy King; Manufacturer: Drip More LLC, CA, United States; USP-grade propylene glycol (PG): USP grade vegetable glycerin (VG) ratio 30:70; United States sourced USP-grade nicotine 0.3%; Food Grade Flavor Extracts) was purchased from a local vendor. A pod device (JUUL; JUUL Labs, CA, United States; 30% PG and 70 %VG) with a 5% nicotine Virginia Tobacco pod was used. Both mod and pod devices were operated using a 4-s puff duration (Meng et al., 2016; Robinson et al., 2015) and a 30-s puff interval protocol under two flow rates (1 LPMF and 1.5 LPM) corresponding to 67 mL and 100 mL puff volumes, respectively.

### 2.3 Carbonyl collection methods

Carbonyls were collected using three collection methods: (Gulsen and Uslu, 2020): two glass impingers in series, each containing 30 mL of an acidified 9 mM DNPH solution (I-I) (CORESTA, 2021), (Zhu et al., 2014) a DNPH cartridge (C) (Khlystov and Samburova, 2016), and (Unger and Unger, 2018) a DNPH-coated glass fiber filter followed by a DNPH cartridge (DF-C) (Son et al., 2020b). To evaluate how carbonyl partitioning between gas and particle phases affects the performance of the carbonyl collection methods, two extra combinations were used: a glass fiber filter followed by two impingers in series (GF-I-I) and a glass fiber filter followed by a DNPH cartridge (GF-C). In all tests, each impinger contained 30 mL of freshly prepared acidified 9 mM DNPH solution.

The number of puffs per sampling media was determined by the carbonyl capacity (formaldehyde equivalent) for each medium and the calibration curve used for analysis. For the I-I and GF-I-I, 30 puffs of e-cigarette aerosol were collected in 10 puff increments with a 5-min pause in between sets. For the DF-C and C methods, five puffs of e-cigarette aerosol were collected. For the GF-C, 10 puffs were collected.

After collection, impinger samples were stored in a fridge (at 4 °C) until analysis. DF and C samples were extracted within 24 h of sampling with 4 mL and 2 mL of acetonitrile, respectively. GF samples were extracted with 5 mL of DNPH solution within 24 h of sampling. The extracts were stored in a fridge (at 4 °C) until analysis. All samples were analyzed for carbonyl compounds within 1 week of collection.

### 2.4 Comparison using known amounts of gas-phase formaldehyde

To exclude any interference from other e-cigarette aerosol components, a test was performed using gas-phase formaldehyde. For this, gas-phase formaldehyde was introduced into a Tedlar chamber (1.8 m^3^) by blowing HEPA-filtered compressed air through a glass impinger containing 259 ± 26 ppm of formalin solution until its complete evaporation and completely filling the bag, achieving 6.1 ± 0.6 ppb formaldehyde concentration in the bag. A mixer was placed inside the chamber to ensure that formaldehyde was properly mixed. To minimize wall losses, sampling was performed quickly after filling the bag, and samples were collected for a short period of time. The media was sampled from a single port in the bag via a 0.5 m Teflon line, which was then split into three lines leading to the impingers in series (I-I) filled with 30 mL of DNPH solution, a DNPH cartridge (C), and a DNPH-coated glass fiber filter, followed by a DNPH cartridge (DF-C). Sample flow rates through each media were controlled by three mass flow controllers (MFC) (Proportional Flow Control Valve, VEMD-L-6-14-20-D21-M5-1-R1-V4, FESTO, Islandia, NY) at 1.0 LPM.

### 2.5 Base material interference

To determine if the carrier liquid material (*i.e.,* PG and VG) interferes with carbonyl measurements using the I-I method, a test was performed in which an aliquot of the impinger solution was taken after the sampling from the Tedlar bag described above, to which 0.67 g of carrier liquid (50:50 PG/VG) was added and shaken vigorously. The amount of carrier liquid added was based on the e-liquid consumption during 30 puffs using a mod at 1 LPM. These samples were treated as the other I-I extracts.

### 2.6 Carbonyl analysis

Collected samples were analyzed using an HPLC-PDA system (Waters ARC 2998 System photodiode array [PDA]) equipped with a Waters XBridge^®^ BEH C18 column (3.0 mm × 75 mm, 2.5 μm). The PDA-acquired spectra in the 210–400 nm range to validate compound identity using its absorption spectrum, with 360 nm used for quantification. A 10-µL sample injection was used, with the mobile phase flow rates being 1.5 mL/min. A detailed description of the mobile phase composition is provided in the Supplementary Material (see Supplementary Table S1). Carbonyl concentrations were quantified using six-point external calibration curves prepared from the certified calibration mixture.

### 2.7 Statistical analysis

Prior to statistical analysis, the HPLC-PDA data were converted to carbonyl emissions (µg/puff) using the concentration in the extract (µg/mL), the extraction volume (mL), and the number of puffs per media. For methods that employ two or more collection methods (I-I, DF-C, etc.), the carbonyl emissions from each method were summed up to obtain total carbonyl emissions. The emissions per puff were then analyzed using the Mann–Whitney U rank test to determine the statistical significance of differences between the collection methods. The statistical test was performed using Python (version 3), with “mannwhitneyu” in SciPy statistical functions. A *p*-value less than 0.1 was used to indicate a significant difference between the collection methods.

## 3 Results

### 3.1 Method evaluation with e-cigarette aerosols

**FIGURE 2:**
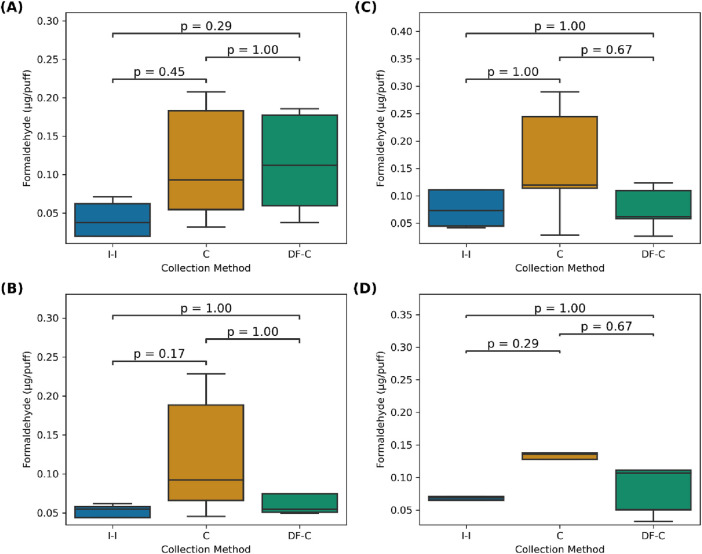
Formaldehyde emissions (µg/puff) per collection method for **(A)** a mod at 1 LPM and **(B)** 1.5 LPM and **(C)** a pod and 1 LPM and **(D)** 1.5 LPM.

**FIGURE 3:**
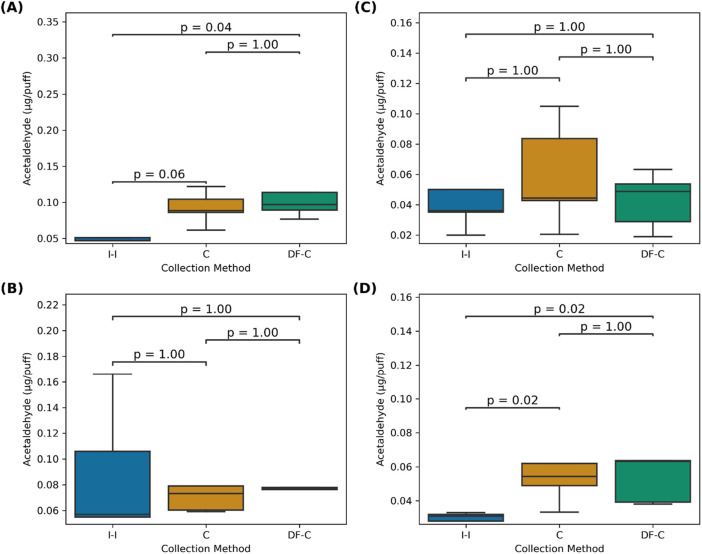
Acetaldehyde emissions (µg/puff) per collection method for **(A)** a mod at 1 LPM and **(B)** 1.5 LPM and **(C)** a pod and 1 LPM and **(D)** 1.5 LPM.

Carbonyl emissions from the pod (*i.e.,* JUUL) and mod devices measured with the three tested methods (C, DF-C, and I-I) at 1 LPM and 1.5 LPM puff flow rates were similar to the ones reported for comparable devices (Beauval et al., 2019; Geiss et al., 2016; Gillman et al., 2016; Reilly et al., 2019; Talih et al., 2019). The results are presented in Table 1 and Figures 2, 3. For both devices, the highest formaldehyde emissions were measured with the C and DF-C methods. The pod emissions measured with the I-I method were slightly lower than those measured with the other two methods; however, for mod devices, the I-I method showed lower emissions.

**TABLE 1:** Carbonyl emissions (µg/puff) for mod and pod devices at 1.0 LPM and 1.5 LPM for all media combinations.

Media	Emission (µg/puff; mean ± SD)							
	Formaldehyde				Acetaldehyde			
	mod		pod		mod		pod	
	1 LPM	1.5 LPM	1 LPM	1.5 LPM	1 LPM	1.5 LPM	1 LPM	1.5 LPM
I-I	0.04 ± 0.02	0.05 ± 0.02	0.10 ± 0.08	0.08 ± 0.04	0.05 ± 0.02	0.09 ± 0.05	0.05 ± 0.04	0.027 ± 0.009
C	0.11 ± 0.08	0.12 ± 0.08	0.2 ± 0.1	0.15 ± 0.08	*0.09 ± 0.02	0.08 ± 0.02	0.06 ± 0.06	*0.06 ± 0.02
DF-C	0.11 ± 0.07	0.10 ± 0.09	0.08 ± 0.04	0.10 ± 0.07	*0.14 ± 0.09	0.09 ± 0.02	0.04 ± 0.02	*0.07 ± 0.04

The gray shaded cells represent the highest value among the tested methods per carbonyl, and the asterisks (*) depict p-value <0.1. Each group has five observations (n = 5).

For example, at 1 LPM, the mod formaldehyde emissions were slightly higher with the C (0.11 ± 0.08 µg/puff) and DF-C (0.11 ± 0.07 µg/puff) method than the I-I method, which indicated 0.04 ± 0.02 µg/puff. However, there were no significant differences compared to the I-I method (*p* = 0.45, *p* = 0.29, respectively). Similarly, at 1.5 LPM, the C (0.12 ± 0.08 µg/puff) and DF-C (0.10 ± 0.09 µg/puff) methods remained higher than the I-I method (0.10 ± 0.09 µg/puff), but these differences were not significant (*p* = 0.17, 1.00, respectively).

At 1 LPM, the mod acetaldehyde emissions measured with the DF-C (0.14 ± 0.09 µg/puff) and C (0.09 ± 0.02 µg/puff) methods were significantly higher (p = 0.04, 0.06, respectively) than those measured with the I-I method (0.05 ± 0.02 µg/puff). At 1.5 LPM, the emissions for the DF-C (0.09 ± 0.02 µg/puff) and C (0.08 ± 0.02 µg/puff) methods remained higher than those measured with the I-I (0.09 ± 0.05 µg/puff) method, although the difference was not significant (*p* = 1.00 for both methods).

Formaldehyde emissions for the JUUL (pod) using the C method were slightly higher at both 1 LPM (0.2 ± 0.1 µg/puff) and 1.5 LPM (0.15 ± 0.08 µg/puff) rates than those measured using the I-I method under the same respective conditions (0.10 ± 0.08, 0.08 ± 0.04 µg/puff), but these differences were not significant (*p* = 1.00; 0.29, respectively). While the C and DF-C results were similar for formaldehyde, at 1 LPM, the pod acetaldehyde emissions measured with the C method were slightly higher (0.06 ± 0.03 µg/puff) than those measured with the DF-C method (0.04 ± 0.02 µg/puff), but the differences were not significantly different (*p* = 1.00 for both) from those of the I-I method (0.05 ± 0.04 µg/puff). However, at 1.5 LPM, the results of both the C (0.09 ± 0.05 µg/puff) and DF-C (0.07 ± 0.04 µg/puff) methods were significantly higher (*p* = 0.02 for both) than those of the I-I method (0.027 ± 0.009 µg/puff).

### 3.2 Particle- and gas-phase carbonyl emissions

To check for the presence of particle-phase carbonyls and to evaluate whether they could affect the performance of the sampling methods, a GF filter was placed before the I-I and C methods to capture particle-phase bound carbonyls, while allowing the following media (I-I and C) to collect only gas-phase carbonyls. Table 2 shows the measured e-cigarette formaldehyde and acetaldehyde emissions using the GF-C and GF-I-I methods. Both methods indicated the presence of a fraction of particle-phase bound carbonyls.

**TABLE 2:** Carbonyl emissions (µg/puff) for mod and pod devices at 1.0 LPM and 1.5 LPM using the GF-C and GF-I-I methods. Each group has five observations (n = 5).

Media	Carbonyls	Emission (µg/puff; mean ± SD, [%])							
		Formaldehyde				Acetaldehyde			
		mod		pod		mod		pod	
		1 LPM	1.5 LPM	1 LPM	1.5 LPM	1 LPM	1.5 LPM	1 LPM	1.5 LPM
GF-C	Total	0.11 ± 0.03	0.10 ± 0.02	0.2 ± 0.1	0.17 ± 0.04	0.07 ± 0.01	0.06 ± 0.01	0.04 ± 0.02	0.04 ± 0.02
	Gas	0.02 ± 0.01 [14%]	0.01 ± 0.01 [15%]	0.06 ± 0.04 [24%]	0.05 ± 0.03 [30%]	0.06 ± 0.01 [91%]	0.05 ± 0.01 [86%]	0.03 ± 0.02 [87%]	0.03 ± 0.02 [90%]
	Particle	0.09 ± 0.03 [86%]	0.08 ± 0.02 [85%]	0.1 ± 0.1 [76%]	0.12 ± 0.05 [70%]	0.01 ± 0.01 [9%]	0.01 ± 0.01 [14%]	0.01 ± 0.03 [13%]	0.01 ± 0.03 [10%]
GF-I-I	Total	0.09 ± 0.03	0.09 ± 0.04	0.10 ± 0.07	0.13 ± 0.07	0.10 ± 0.03	0.12 ± 0.03	0.06 ± 0.01	0.06 ± 0.02
	Gas	0.07 ± 0.03 [75%]	0.07 ± 0.04 [74%]	0.05 ± 0.04 [55%]	0.07 ± 0.05 [54%]	0.09 ± 0.03 [90%]	0.09 ± 0.02 [79%]	0.06 ± 0.01 [100%]	0.06 ± 0.02 [100%]
	Particle	0.02 ± 0.04 [25%]	0.02 ± 0.06 [26%]	0.05 ± 0.08 [45%]	0.06 ± 0.09 [46%]	0.01 ± 0.04 [10%]	0.03 ± 0.04 [21%]	-	-

For total emissions for the e-cigarettes, the mod showed slightly higher emissions using the GF-C method at both flow rates for formaldehyde. For the mod aerosol at 1 LPM, the GF-C method showed 0.11 ± 0.03 µg/puff, while the GF-I-I method showed 0.09 ± 0.03 µg/puff. At the higher flow, the results were similar: 0.10 ± 0.02 µg/puff for the GF-C method and 0.08 ± 0.02 µg/puff for the GF-I-I method. For the JUUL (pod) formaldehyde emissions at 1 LPM, the GF-C method recorded 0.2 ± 0.1 µg/puff, and the GF-I-I method recorded 0.1 ± 0.1 µg/puff. The JUUL at 1.5 LPM showed 0.17 ± 0.04 µg/puff using the GF-C method and 0.13 ± 0.07 µg/puff using the GF-I-I method. However, the differences were not significant.

For the particle phase of the e-cigarette aerosol, for mod and pod at 1 LPM and 1.5 LPM, more of the formaldehyde, acetone, and propionaldehyde were found to be in the particle phase using the GF-C method than the amounts found using the GF-I-I method, but there was no significant difference due to the high variability among methods. The GF-I-I method reported slightly higher particle percentages for acetaldehyde and crotonaldehyde.

### 3.3 Method evaluation using gas-phase formaldehyde

**FIGURE 4:**
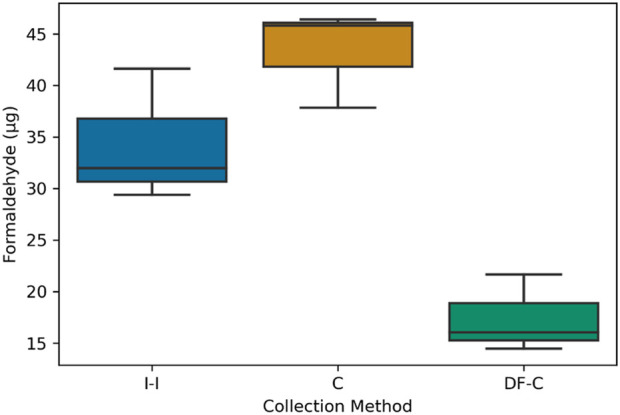
Gas-phase formaldehyde mass (µg) per collection method.

The collection methods were first evaluated using an artificially generated gas-phase formaldehyde that was introduced into a Tedlar bag to achieve a known formaldehyde concentration. The results showed that the C method collected the highest formaldehyde mass (43 ± 5 µg), followed by the I-I method (34 ± 6 µg), and the lowest being the DF-C method (17 ± 4 µg) (Figure 4). The mass of formaldehyde collected with the C method was 29.5% less than expected (61 ± 6 µg). This discrepancy could be attributed to formaldehyde being lost to the walls and tubing of the Tedlar bag.

### 3.4 Interaction of the carrier liquid and carbonyl emissions from the e-cigarette aerosol

During e-cigarette puffing, the aerosol is supersaturated with a gas-phase carrier liquid that is soluble in the DNPH solution used in the impingers. Large quantities of PG and VG can thus be collected in the impingers during e-cigarette testing. Prior to the addition of the carrier base material, a range of 17.04 ± 0.03 µg to 24.87 ± 0.03 µg per sample of formaldehyde was reported. After addition, the amount of formaldehyde was reduced to 21.57 ± 0.03 µg per sample from 14.13 ± 0.03 µg per sample. The results showed that 2.9 ± 0.4 µg of formaldehyde per sample were lost by adding 0.67 g of base material.

## 4 Discussion

This study evaluated the performance of three carbonyl collection media that are frequently used in electronic cigarette studies, namely: 2,4-dinitrophenylhydrazine (DNPH) solution (I-I), a DNPH cartridge (C), and a DNPH filter followed by a DNPH cartridge (DF-C). These collection methods were tested with mod- and pod-type e-cigarettes that represent devices producing different amounts of e-cigarette aerosols.

Based on the results of this study, the existing sampling methods showed similar carbonyl emissions for the pod but not for the mod devices. While our results are based on only two devices and further verification is warranted, the cause for the observed differences could lie in the operating principles of the tested methods and their relative performance when collecting gaseous and particulate components of e-cigarette aerosols. The I-I method used for e-cigarettes was developed by CORESTA (CRM No. 96) and was adapted from a previous method (CRM No. 74) designed for conventional cigarette testing. The aerosol production and aerosol properties differ significantly between conventional tobacco cigarettes and e-cigarettes. In conventional cigarettes, the aerosol is produced by burning/pyrolyzing the tobacco material at temperatures at approximately 700–950 °C (Auer et al., 2017; Jiang et al., 2023), with the aerosol consisting of oxidation and pyrolysis products of the burning material that are solid or tar-like (Paschke et al., 2016). In contrast, an e-cigarette aerosol is produced by evaporation at 100–350 °C of the e-liquid and its consequent recondensation (Mallock et al., 2019). Thus, e-cigarette aerosols consist largely of the main e-liquid constituents—PG and VG—in their liquid form. The liquid nature and chemical polarity of the e-cigarette aerosol make it possible for other polar chemical compounds (such as carbonyls) to dissolve, at least partly, in the aerosol particle phase (Wu et al., 2022). Indeed, it has been reported that a large fraction of carbonyl compounds in e-cigarette aerosols could be in the particle phase (Jiang et al., 2023; Pankow, 2017; Pankow et al., 2018; Wu et al., 2022).

Impingers, such as those used in the CORESTA method, were designed to collect gases, while their collection efficiency for particles is rather poor (Miljevic et al., 2009; Spanne et al., 1999). Thus, impingers could miss carbonyl compounds dissolved in the particle phase of e-cigarette aerosols. DNPH cartridges, on the other hand, are made of a packed bed of DNPH-coated silica beads with a polyethylene filter on both ends, all of which act as a filter capturing both gases and particulates. It is thus likely that the C method performs better than the I-I method in capturing both gas-phase and particle-phase carbonyl compounds. The discrepancy between the cartridge and impinger methods observed in this study generally correlated with the amount of liquid aerosol produced by the device. The larger amount of aerosol particles produced by the mod device could lead to a larger fraction of carbonyls partitioning to the particle phase, which could explain the trend observed in this study because impingers do not collect the particles efficiently. However, due to the significant variability of emissions from puff to puff, this statement cannot be statistically confirmed, and further investigation is needed. These differences between the I-I and C methods could also explain the discrepancies observed between different studies of e-cigarette carbonyl emissions (Khlystov and Samburova, 2018).

The potential interference of dissolved PG and VG with the recovery of carbonyls raises questions about the accuracy of DNPH-based methods in measuring carbonyl emissions in e-cigarette aerosols. We observed that the presence of PG and VG, which is expected to be dissolved in the impinger during e-cigarette aerosol collection, reduces formaldehyde recovery by 17.08%. Some studies have reported that propylene glycol (PG) and vegetable glycerin (VG) can react with aldehydes in solution to form other species, such as hemiacetals (Jensen et al., 2015; Salamanca et al., 2017; Salamanca et al., 2018), which could potentially explain this interference.

The discrepancies between the C and I-I methods, however, cannot be fully attributed to the differences in particle collection efficiencies. The experiments with pure gas-phase formaldehyde showed that the C method reported amounts that were closer to the expected values. The I-I method, on the other hand, showed a lower amount. The DF-C method also showed much lower amounts than expected. While the exact reason for the underestimation by the I-I and DF-C is unclear, one possible explanation could be the losses of formaldehyde to the walls of the impinger and the filter holder that precede the collection on the sorbent media (DNPH solution or DNPH filter). Unlike the C method, both methods have a substantial surface area at the front end of the instruments that is not washed/extracted for analysis. Whether wall losses are the cause of the observed underestimation must be investigated further.

Given the above considerations, the C method appears to be preferable to the I-I method. The I-I method is also more labor intensive and has a lower sensitivity, as the sample is dissolved in a significantly larger volume (30 mL for one impinger vs. 2 mL for a cartridge). On the other hand, the lower sensitivity of the I-I method requires the collection of a larger number of puffs, which could produce a more representative sample of emissions. The largest drawback of the I-I method, however, remains the poor collection efficiency of the particle phase and possible wall losses. This introduces a potentially large uncertainty in comparisons of emissions from different devices that produce different amounts of liquid particles. These findings have important implications for regulatory testing and inter-study comparability. Given that I-I methods are commonly used in regulatory contexts and published research, the systematic underestimation of carbonyl emissions, particularly for high-power devices generating substantial particulate matter, raises concerns about the adequacy of current standardized methods for e-cigarette testing. Discrepancies between methods could contribute to the wide variability in reported carbonyl emissions across studies, complicating risk assessments and regulatory decisions. Harmonization of analytical methods and consideration of device-specific aerosol characteristics may be necessary to ensure reliable inter-study comparisons and appropriate regulatory oversight.

## Data Availability

The raw data supporting the conclusions of this article will be made available by the authors, without undue reservation.

## Abbreviations

C, DNPH cartridge; CORESTA, Cooperation Centre for Scientific Research Relative to Tobacco; DF-C, DNPH filter followed by a DNPH cartridge; DNPH, 2,4 dinitrophenyl hydrazine; E-cigarette(s), Electronic cigarette(s); GF, Glass fiber filter; HPLC, High-performance liquid chromatography; I-I, Impingers in series; I, Impinger; ISO, International Organization for Standardization; L, liter; LPM, liters per minute; MFC, mass flow controller; PDA, photodiode array; PG, propylene glycol; VG, vegetable glycerin (or glycerol).
