# Supplementary material for: Evaluation of carbonyl collection methods in electronic cigarette aerosols
Source: Front Chem. 2025 Nov 26;13:1694858. doi: 10.3389/fchem.2025.1694858 (PMC12689933; doi:10.3389/fchem.2025.1694858)
Supplement: Supplementary file 1 [file Supplementaryfile1.docx]

# Supporting Information

Table S1: Mobile phase gradient percentage for HPLC-PDA sample analysis

|  | Solvent | Mobile Phase Gradient Percentage (%) | Time (min) |
| --- | --- | --- | --- |
| 1 | Water | 65 | 0.0 - 1.5 |
|  | Acetonitrile | 30 |  |
|  | Tetrahydrofuran | 5 |  |
| 2 | Water | 60 | 1.5 – 9.0 |
|  | Acetonitrile | 35 |  |
|  | Tetrahydrofuran | 5 |  |
| 3 | Water | 45 | 9.0 - 10.0 |
|  | Acetonitrile | 50 |  |
|  | Tetrahydrofuran | 5 |  |
| 4 | Water | 65 | 11.0 - 12.0 |
|  | Acetonitrile | 30 |  |
|  | Tetrahydrofuran | 5 |  |

Table S2: Method detection levels for DNPH- Aldehyde adducts (in ng/mL) using the method described above

| Compound Name | Standard Deviation (ng/mL) | LOD (ng/mL) | LOQ (ng/mL) |
| --- | --- | --- | --- |
| Formaldehyde-DNPH | 0.8 | 2.3 | 7.6 |
| Acetaldehyde-DNPH | 2 | 6 | 21 |
| Acetone-DNPH | 3 | 10 | 34 |
| Acrolein-DNPH | 2 | 6 | 19 |
| Propionaldehyde-DNPH | 2 | 6 | 20 |
| Crotonaldehyde-DNPH | 3 | 9 | 30 |
| n-Butyraldehyde-DNPH | 2 | 7 | 24 |
| Benzaldehyde-DNPH | 4 | 12 | 39 |
| Isovaleraldehyde-DNPH | 6 | 19 | 62 |
| Valeraldehyde-DNPH | 3 | 10 | 35 |
| O-Tolualdehyde-DNPH | 3 | 8 | 27 |
| M+P-Tolualdehyde-DNPH | 2 | 5 | 17 |
| Hexaldehyde-DNPH | 8 | 24 | 81 |
| 2,5-Dimethylbenzaldehyde-DNPH | 4 | 11 | 36 |

DNPH – Aldehyde adducts (i.e. Formaldehyde-DNPH, Acetaldehyde-DNPH, Acetone-DNPH, Acrolein-DNPH, Propionaldehyde-DNPH, Crotonaldehyde-DNPH, n-Butyraldehyde-DNPH, Benzaldehyde-DNPH, Isovaleraldehyde-DNPH, Valeraldehyde-DNPH, O – Tolualdehyde-DNPH, M+P- Tolualdehyde-DNPH, Hexaldehyde-DNPH, and 2,5- Dimethylbenzaldehyde-DNPH) were detected with a 360 nm wavelength while full spectrum readings (210 nm – 400nm) were used to confirm the identity of individual compounds. To generate the calibration curve, a certified carbonyl calibration mixture (TO11/ IP-6A Aldehyde/ Ketone – DNPH Mix; Sigma Aldrich, CRM4M7285) was used.
